# Mesenchymal Stromal Cells Cultured in Serum from Heart Failure Patients Are More Resistant to Simulated Chronic and Acute Stress

**DOI:** 10.1155/2018/5832460

**Published:** 2018-04-01

**Authors:** Timo Z. Nazari-Shafti, Zhiyi Xu, Andreas Matthäus Bader, Georg Henke, Kristin Klose, Volkmar Falk, Christof Stamm

**Affiliations:** ^1^Deutsches Herzzentrum Berlin (DHZB), Berlin, Germany; ^2^Deutsches Zentrum für Herz-Kreislaufforschung (DZHK), Partner Site Berlin, Berlin, Germany; ^3^Berlin Institute of Health (BIH), 10178 Berlin, Germany; ^4^Berlin Center for Regenerative Therapies (BCRT), Berlin, Germany

## Abstract

Despite regulatory issues surrounding the use of animal-derived cell culture supplements, most clinical cardiac cell therapy trials using mesenchymal stromal cells (MSCs) still rely on fetal bovine serum (FBS) for cell expansion before transplantation. We sought to investigate the effect of human serum from heart failure patients (HFS) on cord blood MSCs (CB-MSCs) during short-term culture under regular conditions and during simulated acute and chronic stress. Cell survival, proliferation, metabolic activity, and apoptosis were quantified, and gene expression profiles of selected apoptosis and cell cycle regulators were determined. Compared to FBS, HFS and serum from healthy donors (CS) showed similar effects by substantially increasing cell survival during chronic and acute stress and by increasing cell yields 5 days after acute stress. Shortly after the termination of acute stress, both HFS and CS resulted in a marked decrease in apoptotic cells. Transcriptome analysis suggested a decrease in TNF-mediated induction of caspases and decreased activation of mitochondrial apoptosis. Our data confirm that human serum from both healthy donors and heart failure patients results in increased cell yields and increased resistance to cellular stress signals. Therefore, we consider autologous serum a valid alternative to FBS in cell-based therapies addressing severe heart disease.

## 1. Introduction

**Figure 1:**
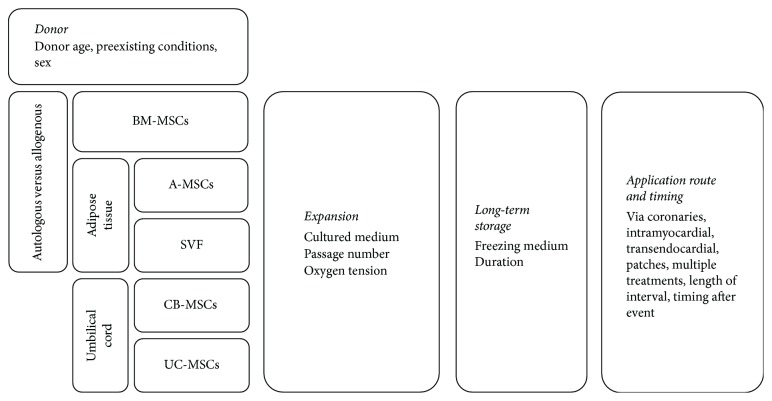
Examples of different variables that may influence the efficacy and efficiency of mesenchymal stromal cell transplantation in the setting of cardiovascular regeneration. BM-MSCs: bone marrow MSCs; A-MSCs: adipose tissue-derived MSCs; SVF: stromal vascular fraction; CB-MSCs: cord blood MSCs; UC-MSCs: umbilical cord matrix MSCs.

To date, there have been several clinical studies investigating the use of mesenchymal stromal cells (MSCs) for treatment of cardiovascular disease. Based on the findings of Kawada et al. [1] and Chen et al. [2] demonstrating the regenerative potential of bone marrow MSCs (BM-MSCs), various BM-MSC preparations were transplanted into infarcted myocardium. It is currently assumed that, independent of their source, MSCs can prevent myocardial remodeling following acute myocardial infarction (AMI) via paracrine secretion of gene expression modulating and trophic factors [3, 4]. In addition, a number of trials tested the myocardial injection of MSCs in the setting of chronic ischemic cardiomyopathy, hoping to induce reverse myocardial remodeling [5–7]. Multiple factors influence the outcome of cellular therapy, and there have been attempts to address those recently **(**Figure 1), including the timing (TIME-Study [8]) and frequency of MSC administration, the route of administration, and the source of stromal cells. There have also been attempts to modify MSCs prior to transplantation to improve their engraftment efficacy and clinical effects (i.e., C-CURE [9], CHART-1 [10], and IMPACT-DCM [11]). Cells transplanted into damaged myocardium are exposed to acute and chronic stress within a hypoxic and malnutritioned microenvironment. Additionally, MSC culture conditions greatly influence their stress responses after transplantation. It has been well established that cell culture introduces DNA damage and functional changes in MSCs [12]. The most common cell culture media used today contain heat-inactivated fetal bovine serum (FBS). However, in addition to its regulatory difficulties, FBS also introduces such variables in MSC products that are difficult to control, causing dysregulation of cell cycle and metabolism [13]. Consequently, many groups have been working on formulating a serum-free culture media with recombinant growth factors [14, 15]. However, some controversy around the effect of serum-free culture medium on MSC function remains [16–18]. An alternative approach to eliminate the issues surrounding FBS is the use of autologous recipient serum for MSC *in vitro* culture [19, 20]. Other groups, including our team, have previously shown that serum from patients with heart failure can impair MSC function [21]. In fact, a retrospective analysis of patients with chronic heart failure (CHF) treated with BM-MSCs that were cultured in either FBS or autologous serum demonstrated less variance in population doublings in the FBS group [22].

In the current study, we sought to investigate whether the short-term incubation of a “virginal” model cell product with human serum of CHF has an immediate effect on cell proliferation and metabolism. We chose cord blood-derived MSCs (CB-MSCs) as a model cell type, since CB-MSCs are more proliferative, not senescent, and have not been subjected to exogenous noxae [23]. CB-MSCs show no sign of DNA damage and telomere dysfunction at the time of isolation [24–26]. Additionally, they do not express HLA on their surface, facilitating potential allogenic applications [27]. We assume that, by using model cells free from intrinsic pathology, changes in biologic behavior should solely reflect the impact of the media composition.

## 2. Methods

### 2.1. Clinical Trial Analysis

A literature search on MEDLINE and clinicaltrials.gov was conducted to identify clinical trials testing MSCs for cardiovascular regeneration in the past five years. The search was limited to study protocols or results published between May 1, 2012, and May 31, 2017. A combination of terms for mesenchymal stem cells (“MSC,” “mesenchymal cells,” “bone marrow cells,” “adipose-derived stem cells,” “umbilical cord/blood MSC,” and “stromal vascular fraction”) and disease-related keywords (“cardiovascular disease,” “acute/myocardial infarction,” or “congestive/heart failure,” and “ischemic/dilated cardiomyopathy”) was used to identify relevant trials. Vocabulary and syntax were adjusted across databases. Each study protocol was screened for cell type used and, if applicable, for cell culture medium formulation used for ex vivo expansion of cells (Table 1).

### 2.2. Study Population

In accordance with the Declaration of Helsinki, this study was approved by the Ethics Committee of Charité-Universitätsmedizin Berlin, Berlin, with informed consents signed by all patients (*n* = 12) and volunteers (*n* = 12) (Table 2). A thorough medical history of all patients was obtained, and all current medications were documented. We included patients aged 64 ± 3 years with ischemic cardiomyopathy (ICMP), who had an average left ventricular ejection fraction of 22 ± 2% at the time of blood collection. Two-thirds of the patients had a history of past myocardial infarction, and all patients suffered from heart failure symptoms with New York Heart Association functional class III or IV. Patients with acute or recent myocardial infarction were not included in this study; the average time elapsed since the infarct was 8 ± 3.4 years. Control serum was collected from healthy volunteers aged 54 ± 1.6 years (*p* < 0.05 versus CHF patients) without a history of cardiovascular disease.

### 2.3. Serum Extraction

Venous blood was collected in S-Monovettes® (Sarstedt, Nümbrecht, Germany) by using the BD Vacutainer Safety-Lok blood collection set (BD Medical, Heidelberg, Germany). Whole blood samples were left undisturbed at room temperature for 30 minutes and then centrifuged at 3500*g* for 15 minutes at 4°C to remove the clot and all remaining cellular particles. Serum supernatants were then sterile filtered, aliquoted, flash frozen in liquid nitrogen, and then stored at −80°C for later use. Serum from each donor (*n* = 12) was tested individually on CB-MSCs to account for possible patient-specific confounding factors. CHF is associated with interstitial and intravascular volume retention, and patients usually show relative hyperproteinemia which could influence the quantitative bioactivity of heart failure serum (HFS) as compared to control serum (CS). Accordingly, the total protein concentration in CS was significantly higher than that in HFS (5.9 g/dL in CS, 5.5 g/dL in HFS, *p* = 0.04). We noted that the lowest serum protein concentration in one of our heart failure patients (4.6 g/dL) was within the range of protein concentration found in our FBS sample (4.5 g/dL). Since we hypothesized that differences in serum impact on cells arise from its bioactive contents, that is, cytokines, exosomes, short nucleotides, or other trophic factors, we decided to eliminate the variance in total protein concentration. In order to minimize this systematic error, serum concentrations were normalized concentration within our samples to 4.5 g/dL, by dilution with Dulbecco's phosphate-buffered saline (DPBS) containing Ca^2+^ and Mg^2+^ (Life Technologies, Darmstadt, Germany). Total protein concentration of each serum sample was quantified using the Pierce BCA Protein Assay Kit (Thermo Fisher, Waltham, MA, USA).

### 2.4. *In Vitro* Cultivation of Human CB-MSCs

Human CB-MSCs were provided by courtesy of Dr. K. Bieback, who isolated them from umbilical cord blood with the mothers' consent and approval of the local ethics committee and expanded the cells based on a previously published protocol [28]. Prior to experimentation, the CB-MSCs were thawed, washed, and expanded in 1 g/L glucose DMEM with 10% FBS, under antibiotic protection with 1% streptomycin/penicillin (all from Life Technologies, Carlsbad, CA, USA). We initially tested the effects of FBS from three different lots on proliferation, immunophenotype, and trilineage differentiation capacity of CB-MSCs. No difference was detected, and thus, FBS from one lot was used in all experiments (Life Technologies, Lot 41A1513K). Cells were seeded at 800–1000 cells/cm^2^ in T175 flasks and cultured under 21% O_2_ and 5% CO_2_ at 37°C. Partial media changes were performed every 3 days. All cultured cells were screened for the presence of mycoplasma (MycoAlert™ assay, Lonza, Walkersville, MD, USA). All experiments were performed on cells between passages 4 and 6. CB-MSC phenotype and their ability to differentiate into nonhematopoietic cell types have been repeatedly confirmed in previous experiments by our group [21].

### 2.5. *In Vitro* Models for Acute and Chronic Stress on CB-MSCs

#### 2.5.1. Chronic Stress (ChS)

**Figure 2:**
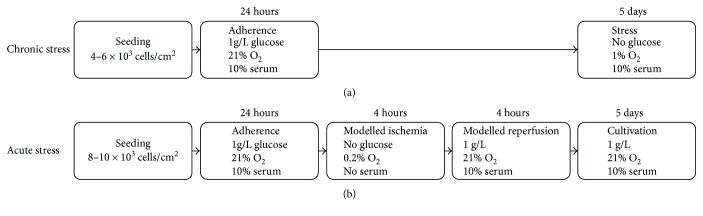
Flow chart depicting the experimental setup for the *in vitro* models of simulated chronic (a) and acute (b) stress. FBS, HFS, or CS was used as a serum supplement where indicated. In selected experiments, IL-6 was added to regular cell culture media supplemented with FBS.

Glucose deprivation in a setting of low oxygen tension triggers oxidative stress in MSCs [29, 30]. To test the response of CB-MSCs to continued stress in culture with either human serum or FBS, we designed an experimental setup where CB-MSCs were cultured under low oxygen tension and glucose deprivation for 5 days (Figure 2(a)). CB-MSCs were seeded at a density of 4–6 × 10^3^ cells/cm^2^ into 96-well plates (Greiner Bio-One, Frickenhausen, Germany). After 24 hours of incubation under standard cell culture conditions to allow for attachment (day 0), cells were deprived of glucose and provided with either 10% human serum or FBS in their culture media and cultivated for 5 days at 1% O_2_. In parallel, as a control condition, cells were kept under regular culture conditions with the exception of supplementing the media with FBS, CS, or HFS. Culture media was changed every other day.

#### 2.5.2. Acute Stress (AcS)

To test how CB-MSCs cultured with different sera behave in response to an acute stress trigger (AcS), we chose an *in vitro* model of “simulated ischemia reperfusion injury” where the cells are deprived of glucose, serum, and oxygen (0.2% O_2_) for 4 hours and then transferred back into regular culture conditions (Figure 2(b)). This setup was designed to test the immediate survival of cells after simulated ischemia, with the use of either CS, HFS, or FBS during their oxygenation phase. All oxygen-deprivation studies were performed in a hypoxic incubator (Binder, Tuttlingen, Germany). Cells were seeded at 8–10 × 10^3^ cells/cm^2^ and cultivated with FBS and 1 g/L glucose for 24 hours at normal cultivation conditions. Then, cells were exposed to hypoxia (0.2% O_2_) as well as serum and glucose deprivation for 4 hours, followed by 4 hours of simulated reperfusion in normal culture settings. The proliferative capacity of CB-MSCs (or recovery) during the first 5 days after the acute stress trigger was tested in culture with the different sera (10% CS, HFS, or FBS).

### 2.6. Metabolic Activity, Cell Counts, and Proliferation Assays

One part of 3-(4,5-dimethylthiazol-2-yl)-5-(3-carboxymethoxyphenyl)-2-(4-sulfophenyl)-2H-tetrazolium (MTS) and phenazine methosulfate (PMS) solution was added to 5 parts of culture media, and the cells were incubated for 4 hours at 37°C. Absorbance (OD) was measured at 490 nm and 650 nm as a reference wavelength. Subsequently, nuclei were stained with Hoechst 33342 (Life Technologies) in the dark for 20 minutes at room temperature and washed with DPBS. Cell numbers were counted in the Operetta High-Content Imaging System (PerkinElmer, Rodgau, Germany) at 380 nm excitation and 445 nm emission. Cell survival rate after AcS is depicted as the percentage of initially plated cells. Cell survival rate is depicted as the percentage of cells counted after AcS, as compared to the percentage of cells plated. BrdU incorporation was used (Roche, Mannheim, Germany) to quantify cell proliferation. Cells cultured in 96-well plates were incubated with BrdU labeling solution at 37°C for 4 hours, and the following steps were performed without interruptions according to the manufacturer's instructions. Absorbance was measured in the ELISA reader (Molecular Devices GmbH, Biberach an der Riss, Germany) at 370 nm with the reference wavelength set to 492 nm.

### 2.7. Apoptosis Detection Assays

Two different assays were performed to quantify apoptosis. Fluorochrome-labeled inhibitors of caspases (FLICA) assays were used to detect caspase activity in CB-MSCs. The polycaspase probe (SR-VAD-FMK), provided by ImmunoChemistry Technologies (LLC, Bloomington, MN, USA), recognizes all the different types of activated polycaspases. Cells were incubated with the polycaspase probe for 45 minutes at 37°C, under gentle agitation every 10 minutes. Then, nuclear counterstaining with Hoechst 33342 was performed. Cells were then scanned with the Operetta High-Content Imaging System at 570 nm and 380 nm excitation and 615 nm and 445 nm emission wavelengths. The data were analyzed using the Columbus software (PerkinElmer). Additionally, the differential detection of late and early stages of apoptosis was performed by FITC-Annexin V and ethidium homodimer III (EthD-III) staining, using a kit provided by Promokine (PromoCell GmbH, Heidelberg, Germany). CB-MSCs were harvested after the AcS and stained according to the manufacturer's protocol. Cells in normal culture were used as negative control. CB-MSCs treated for 24 hours with 200 *μ*M H_2_O_2_ were used as Annexin V-positive control, while cells incubated on ice after 10 minutes of 65°C warm water bath were used as ethidium homodimer III-positive control. FITC- and/or EthD-III-positive cells were then quantified using the MACSQuant VYB (Miltenyi Biotec GmbH, Bergisch Gladbach, Germany) and analyzed with FlowJo 10 (FlowJo, LLC, Ashland, OR, USA).

### 2.8. Semiquantitative Real-Time PCR

Total RNA was isolated using the NucleoSpin RNA isolation kit (Macherey-Nagel, Düren, Germany). The purity and integrity of isolated RNA were determined by spectrophotometry and gel electrophoresis. The RNA samples from biological replicates were pooled before cDNA synthesis to account for biological variation [26]. cDNA was synthesized by reverse transcription using the qScript SuperMix (QuantaBio, Beverly, Massachusetts, USA). The expression of 96 genes associated with apoptosis and cell cycle pathways was tested using RT-PCR array kits from http://realtimeprimers.com (Elkins Park, Philadelphia, USA), using their recommended amplification protocol. All the CT values were normalized to mRNA expression of HPRT1. We collected total RNA after 1 day of cultivation with FBS, CS, and HFS and then 4 hours after AcS, as well as 1, 3, and 5 days after AcS under continued cultivation with either FBS, CS, and HFS. The fold change of the mRNA expression before AcS of 119 genes was included in a heatmap, using average-linkage hierarchical clustering. The grouping of the genes in the heatmap was determined by calculating the distance among the groups by the Spearman rank correlation method.

### 2.9. Statistics

All results are shown as mean ± SEM. Unless stated otherwise, experiments were performed with group sizes of at least four control samples (FBS) and 12 human serum samples. Normal distribution and homogeneity of variances (Levene's test) were tested. With a normal distribution provided, ANOVA with Tukey's post hoc analysis was done; otherwise, the Kruskal-Wallis test or Mann–Whitney *U* test was applied to test for differences between groups. In cases where Levene's test showed heterogeneity, Welch ANOVA and Games-Howell post hoc tests were performed. The differences were considered statistically significant at *p* < 0.05. Repeated measures ANOVA was performed along with the experiments that had three or more time points. Statistical analyses were performed using the IBM SPSS Statistics software Version 22 (IBM Corporation, Somers, NY, USA).

## 3. Results and Discussion

### 3.1. Sera Used in Clinical MSC Trials

**Figure 3:**
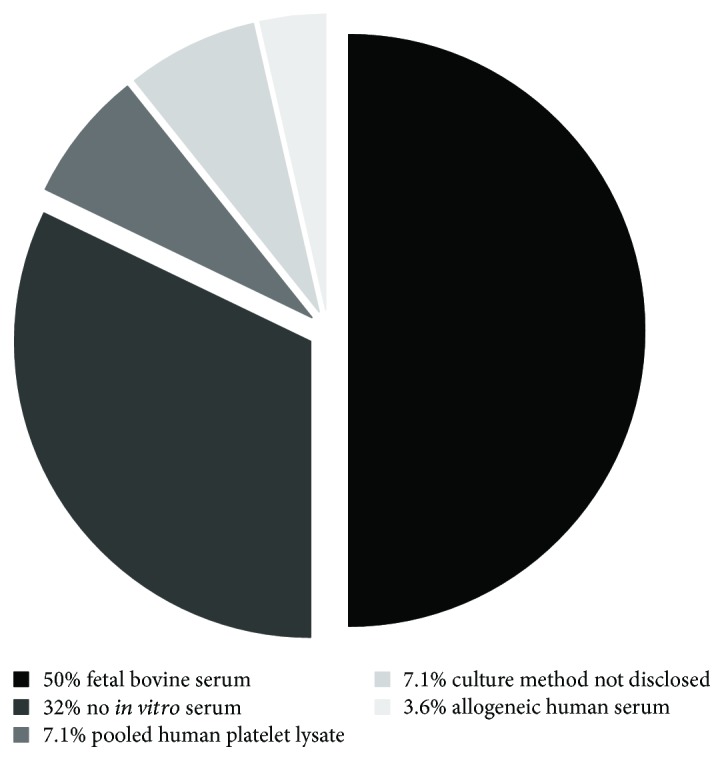
Analysis of culture media supplements used in clinical trials in the past five years (cutoff 07/2012) utilizing MSCs in cardiovascular regeneration. 28 trials were identified, of which only three used human blood products for media supplementation (1 with allergenic serum and 2 with platelet lysate). Trials without *in vitro* expansion (*n* = 9) were also included. Overall, 50% of the clinical trials relied on FBS as a supplement (*n* = 14). In two reports, the exact formulation of the culture media could not be identified based on the information provided.

As shown in Figure 3 and Table 1, the results from 28 trials using MSCs for cardiovascular cell therapy were published within the past five years. The therapeutic effect of MSCs was tested in patients with congestive heart failure (61%), acute myocardial infarction (29%), or coronary artery disease without clinical signs of myocardial infarction (11%) (Table 1). In 50% of the studies, MSCs were expanded in FBS-containing cell culture medium prior to transplantation into patients. Only three clinical trials utilized either allogeneic or autologous human blood products. In two trials, allogeneic pooled human platelet lysate was used (C-CURE trial, NCT00810238; CHART-1, NCT01768702) to expand autologous BM-MSCs prior to implantation. In one trial (HUC-HEART trial, NCT02323477), human serum from healthy donors with the blood group AB was used to expand human umbilical cord MSCs. In one-third of the published trials, cell products that did not require any cell culture after isolation and prior to transplantation were used. This includes trials utilizing adipose-derived stromal vascular cells and bone marrow-derived mononuclear cell preparations.

### 3.2. Human Serum Improves Proliferative Capacity of CB-MSCs

**Figure 4:**
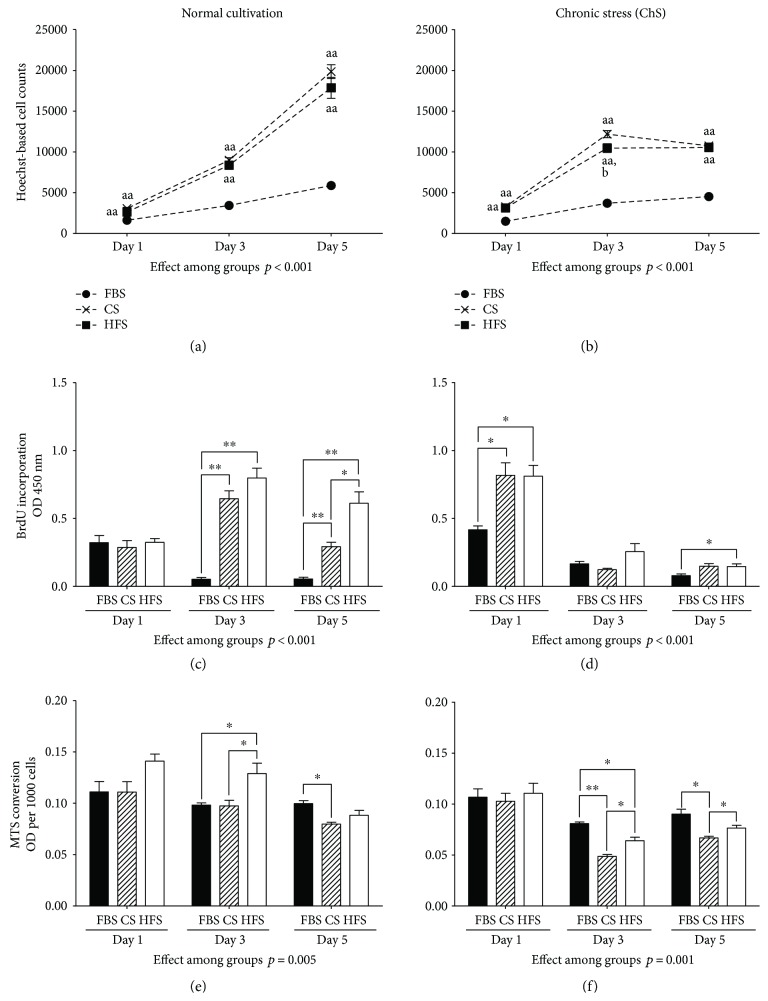
CB-MSC behavior during 5 days of incubation under normoxia and regular glucose supplementation (a, c, e) and under “chronic stress” (1% O_2_ and glucose deprivation) (b, d, f). Shown are cell numbers (a, b), proliferation via BrdU incorporation (c, d), and metabolic activity via MTS conversion (e, f). There were significant proproliferative effects with human sera (CS and HFS). Compared to CS, HFS elevated cellular metabolic activities in both conditions. ^∗^*p* < 0.05 and ^∗∗^*p* < 0.01. ^aa^*p* < 0.01 compared to corresponding FBS; ^b^*p* < 0.05 HFS groups compared to corresponding CS groups.

For clinical use of CB-MSCs, the use of an autologous serum may reduce the regulatory burden and risks of foreign pathogens in cultured cells. However, heart failure serum factors may negatively influence cells [21]. Therefore, we tested whether the presence of heart failure in serum donors affects the proliferation profile of CB-MSCs during short-term cultivation. We previously showed that the cultivation of CB-MSCs with human serum neither from healthy individuals nor from heart failure patients affects the overall morphology or immunophenotype of the cells [21]. In the current series of experiments, individual human serum (CS and HFS) supplementation resulted in a greater variability of the CB-MSC growth rate than FBS, which is a standardized, pooled tissue culture supplement (Figure 4(a)). Pooled human serum may have produced less variation but since we aimed to test whether the use of autologous HF serum is feasible for MSC cultivation, pooled serum would not reflect the potential translational scenario. Cell culture with CS and HFS resulted in significantly higher cell yields compared to FBS. (Figure 4(a)). The significant increase in DNA synthesis in HFS and CS compared to FBS treatment indicates that the higher cell yields are a result of increased cell proliferation (Figure 4(c)). Interestingly, CB-MSCs subjected to HFS maintained a high BrdU incorporation rate throughout day 5, whereas the proliferation rate declined in CS-treated cells on day 5, when they reached confluency, which in itself is an inhibitor of cell proliferation of MSCs. Similarly, MTS conversion rate on day 3 was higher with HFS than with CS (0.13 ± 0.01, as compared to 0.1 ± 0.02 with CS, *p* = 0.03) (Figure 4(e)), indicating increased metabolic activity and/or higher cell numbers. However, this effect was no longer detectable by day 5. Overall, our data confirm that the proliferation profiles of CB-MSCs are significantly better under the cultivation with human serum, regardless of whether the donor suffered from CHF or not. Kubo et al. recently reported that autologous serum from older donors with CHF prolonged the population doubling times of BM-MSCs, as compared to FBS and serum from donors without CHF [22]. “Aged” serum was also found to negatively affect mesenchymal stem cells in mice [31], and aging satellite cells could be rejuvenated when exposed to a young serum [32, 33]. Although we cannot eliminate the possibility that donor age affects the proliferation profile of CB-MSCs, we did not observe a clearly negative serum effect that could be attributed to age. Compared to the “age” of the CB-MSCs (neonatal), the difference in CS and HFS donor age (54 versus 64 years) may be too small to elicit a relevant functional response.

### 3.3. *In Vitro* “Chronic Ischemia” Model

As mentioned above, cells transplanted into ischemic or scarred myocardium are exposed to a poorly vascularized microenvironment, with low tissue oxygenation and impaired nutrition supply. MSCs heavily depend on a glycolytic metabolism under physiological conditions [30], and dependence on glycolytic pathway further increases in lower oxygen tension [29]. We therefore exposed the cells to a model of chronic “ischemic” stress by removing glucose from the cell culture media and reducing the oxygen supply during culture to 1% O_2_. Combined with glucose starvation, this model significantly inhibited CB-MSC proliferation regardless of the source of the supplemented serum (Figure 4(b)). However, CB-MSCs cultured with human serum (both CS and HFS) initially maintained significantly higher BrdU incorporation than cells in FBS (Figure 4(d)). In all groups, on days 3 and 5, CB-MSCs cultured under glucose deprivation went into cell cycle arrest with almost no detectable BrdU incorporation. Interestingly, cells cultivated with FBS maintained higher levels of metabolic activity, when compared to those cultured with human CS or HFS (Figure 4(f)). This may indicate that the suppression of metabolic activity, a presumably protective measurement during stress, was more effective in CS and in HFS-treated CB-MSCs [34, 35].

### 3.4. *In Vitro* “Acute Ischemia” Model

**Figure 5:**
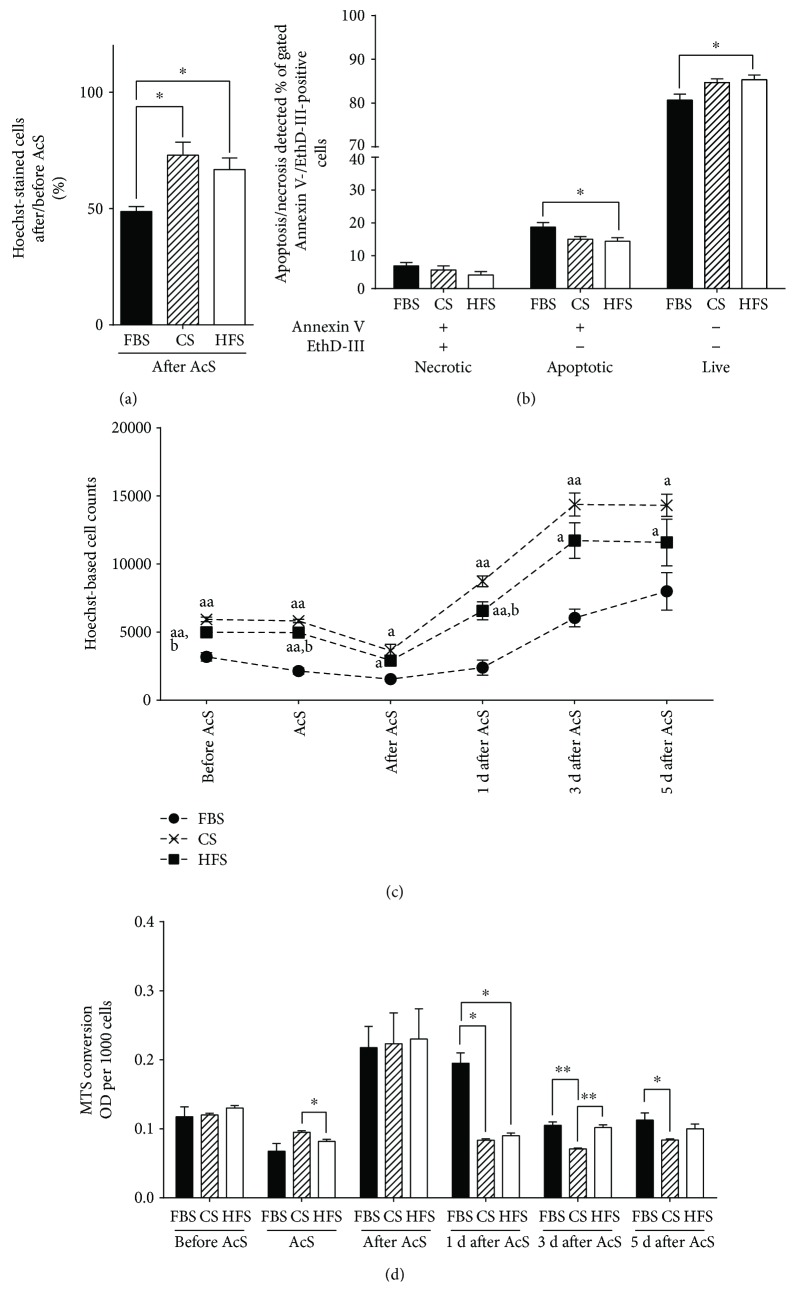
Overview of survival and recovery of CB-MSCs after HFS and CS pretreatment and AcS. Survival rates (a) were calculated by normalizing to the cell numbers before AcS. Percentages of apoptotic, necrotic, and live cells (b) were quantified by flow cytometry. CB-MSC recovery was assessed by cell counting for five days (c). Metabolic activity was measured throughout days 0, 1, 3, and 5 after AcS (d). ^∗^*p* < 0.05 and ^∗∗^*p* < 0.01; ^a^*p* < 0.05 and ^aa^*p* < 0.05 compared to the corresponding FBS groups and ^b^*p* < 0.05 HFS groups compared to the corresponding CS groups.

Because cells are exposed to acute stress during the process of transplantation, we designed an *in vitro* model mimicking ischemia/reperfusion injury. In our preliminary experiments, we established that the combination of 4 hours of hypoxia at 0.2% O_2,_ combined with glucose and serum deprivation and followed by reoxygenation in normoxic culture conditions with serum and glucose, is sufficient for reproducibly inducing significant cell loss. Immediately following simulated acute ischemia, we observed that the rescue of cells cultured with HFS (66.8% ± 5%) and CS (72.9% ± 5.6%) was significantly higher than the cells treated with FBS (48.7% ± 2.1%) (*p* = 0.013 versus *p* = 0.004, resp.) (Figure 5(a)). Moreover, when we performed Annexin V and EthD-III stainings after AcS, we found that the number of apoptotic cells was significantly lower in HFS than in cells treated with FBS (HFS, 14.4% ± 1.1% versus FBS, 18.8% ± 1.4%, *p* = 0.04) (Figure 5(b)). During the reoxygenation period, cells incubated with CS or HFS recovered much faster and showed significantly higher proliferation rates (Figure 5(c)). As expected, AcS led to a significant decrease in metabolic activity in all treatment groups (Figure 5(d)). After the reoxygenation/recovery period, MTS conversion rates were markedly increased compared to baseline, independently from which serum was used. In CB-MSCs cultured with HFS or CS, MTS conversion rates returned to pre-AcS levels at day one. In FBS-cultured cells, metabolic activity did not return to baseline until day three. The increase in metabolic activity during the recovery period could be explained by the sudden increase in reactive oxygen species (ROS) during simulated reperfusion and the increased ATP demand to overcome the imbalance in intracellular calcium homeostasis induced by simulated ischemia [36]. The faster recovery to baseline metabolic rates further supports the hypothesis that HFS and CS protect CB-MSCs during acute cellular stress (Figure 5(d)).

### 3.5. The Role of Interleukin-6 as a Representative Heart Failure Serum Factor

**Figure 6:**
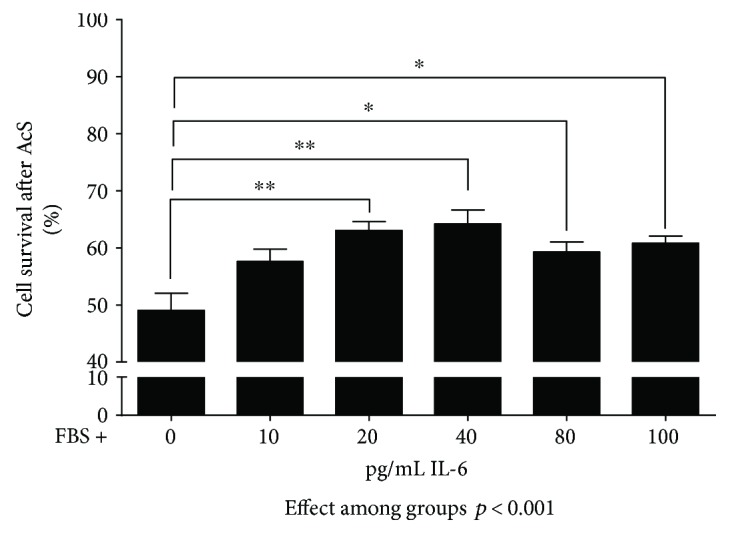
Preliminary experiments to establish the appropriate IL-6 concentration for *in vitro* testing. Cells were seeded at a density of 3 × 10^3^/cm^2^. After 1 day in cell culture, cells were subjected to 4-hour simulated acute “ischemia” and 4-hour reoxygenation (AcS). Cell numbers were quantified by Hoechst-based nucleated cell counts. Cell survival was calculated by dividing cell numbers from experimental groups by control group. ^∗∗^*p* < 0.001 and ^∗^*p* < 0.05.

**Figure 7:**
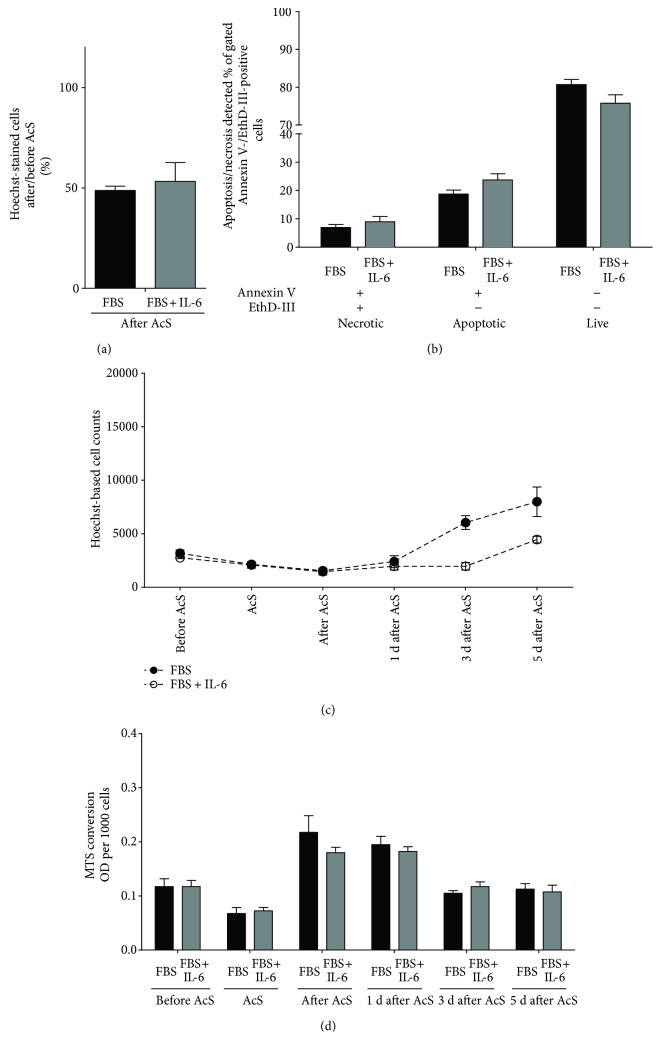
Survival and recovery of CB-MSCs after treatment with 40 pg/mL of human recombinant IL-6 protein and AcS. Survival rates (a) were calculated by normalizing to the cell numbers before AcS. Ratios of apoptotic, necrotic, and live cells (b) were quantified by flow cytometry. Cell numbers (c) and metabolic activity (d) were measured one day before AcS. Measurements were repeated 4 hours after AcS and at days 1, 3, and 5 after AcS.

In our previous work, we showed that IL-6 titers are significantly higher in sera from patients with CHF [21]. IL-6 facilitates proinflammatory signaling in the setting of acute and chronic injury, can also initiate apoptosis in tissues exposed to inflammation [37], and has been associated with cardiomyocyte hypertrophy and myocardial dysfunction. Therefore, we studied whether supplementation of FBS media with recombinant human IL-6 has an effect on growth rate, metabolic activity, and/or apoptosis of CB-MSCs in the acute injury model. IL-6 is vital for the innate and adaptive immune system [38] and has been shown to be present in increased concentrations in sera from heart failure patients [21]. IL-6 was shown to increase HIF-1a through the IL-6-STAT3-HIF1 signaling pathway [39], and HIF-1a exhibits a protective effect on cells under hypoxic stress [40]. Also, IL-6 was shown to increase cell survival under hypoxia and other different conditions [41, 42]. Thus, we hypothesized that pretreatment of IL-6 increases the resistance of CB-MSCs to “ischemic” stress. We first performed preliminary experiments for treating CB-MSCs with increasing concentrations of recombinant human IL-6, followed by exposure to “acute stress.” As shown in Figure 6, a concentration of 40 pg/mL IL-6 indeed improved the survival of CB-MSCs when compared to the FBS control. However, the protective effects proved not to be statistically significant in the subsequent serial experiments over time (Figure 7). There was no significant difference in survival rates between FBS plus IL-6 and FBS immediately at the end of “simulated ischemia” (Figure 7(a)). Necrotic and apoptotic cells did not decrease, and live cells did not increase based on Annexin V/EthD-III stainings (Figure 7(b)). Similarly, there was no evidence of an improved MTS conversion rate in response to IL-6 (Figure 7(d)). In fact, total cell counts appeared to be lower in the presence of IL-6 on days 3 and 5 after AcS (Figure 7(c)).

### 3.6. Transcriptional Profiling

**Figure 8:**
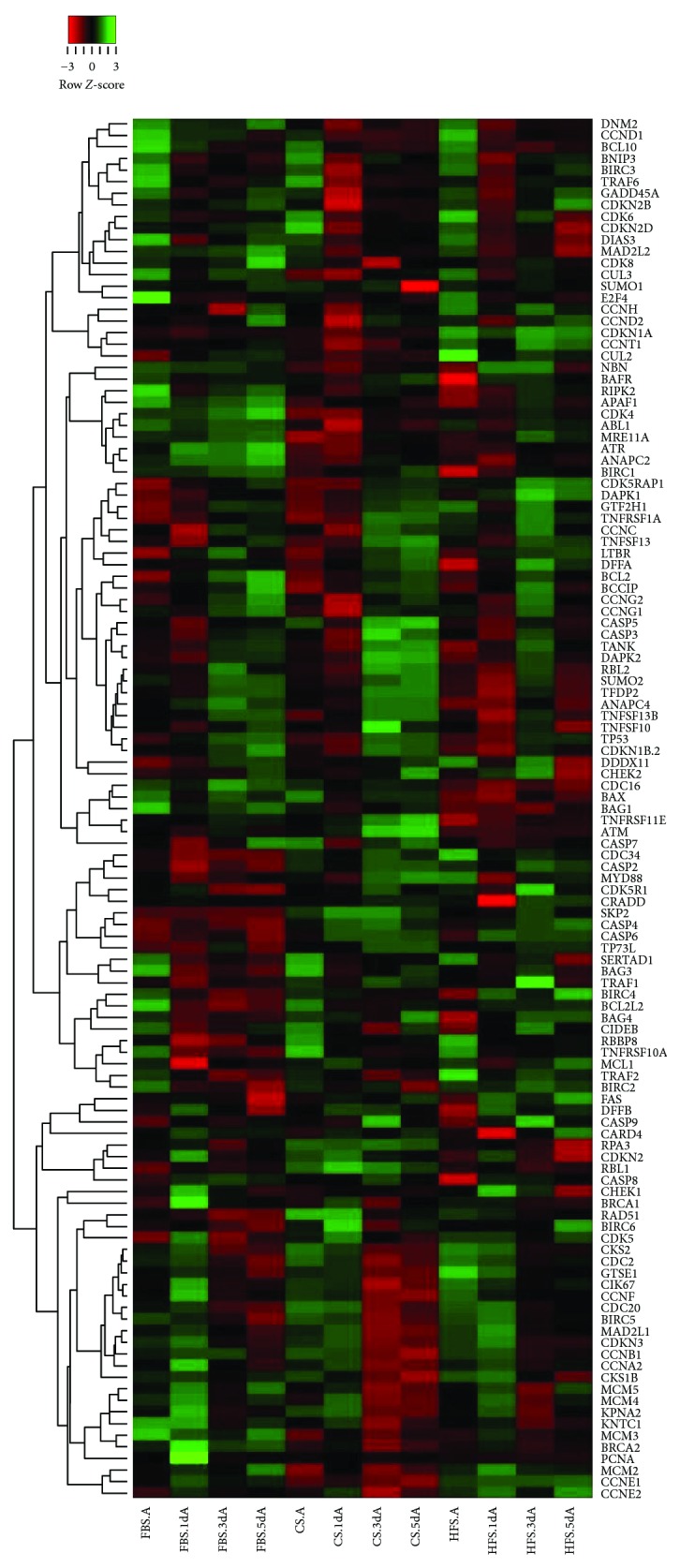
Gene regulation of CB-MSCs under FBS, HFS, and CS cultivation through 5 days of recovery after AcS. The fold changes of mRNA expression relative to unexposed cells of 119 genes were included in this heatmap, which was constructed by using average linkage hierarchical clustering. Gene list was sorted, and distances among groups were calculated by Spearman rank correlation method. Relative regulation levels were represented by different colors, which reflected the row *z*-score shown in the red-green key.

In order to better understand the pathways regulating apoptosis and cell cycle that are affected during acute stress under the influence of the different sera, we performed an RT-PCR array with panels of mRNAs coding for the proteins involved in apoptosis and cell cycle pathways (Figure 8). Our analysis suggests a differential expression of mRNAs related to the DNA damage response and induction of cell cycle arrest during the time course after acute injury. While the expression patterns remained similar in all the groups even 4 hours after AcS, proapoptotic genes like BNIP3 (BCL2-interacting protein 3) and antiproliferative genes like p15 (CDKN2B, cyclin-dependent kinase inhibitor 2B) were downregulated during the 5 days following AcS. Downregulation of BNIP3 in CB-MSCs during hypoxic preconditioning has been associated with an increased survival in response to stress via activation of BCL2 (B-cell lymphoma 2), which also showed an increased expression in HFS- and CS-treated cells on days 1, 3, and 5 [43]. Interestingly, CB-MSCs cultured in HFS, CS, and FBS alike demonstrated an increased response in growth arrest and DNA damage-inducible protein upregulation (GADD45A, growth arrest, and DNA damage-inducible 45 alpha) 4 hours after AcS. Consequently, the expression levels decreased in the CS and HFS cells, while the expression levels remained at a higher level than before AcS in the FBS-treated cells. The GADD45 family is a key player in stress-induced cell arrest, for example, through oxidative stress or DNA damage [44]. The lower levels of cell proliferation, observed in CB-MSCs treated with FBS, may in part be explained by the continued GADD45A-induced cell arrest after AcS.

As mentioned in the previous section, the increased yield of CB-MSCs cultured with human serum could only in part be explained by the increased proliferation. After AcS, we observed significantly lower cell loss and decrease in apoptosis in the cells treated with human serum. The array analysis confirmed the activation of expression of multiple antiapoptotic mRNAs in HFS- and CS-treated cells, especially at the early time points after AcS. The most prominent was an increase in the expression of TRAF1 (TNF receptor-associated factor 1) and TRAF2 as well as mRNA expression of the BIRC-family. By binding the TRAF1/2 complex to the intracellular domain of the TNF receptor, TRADD (tumor necrosis factor receptor type 1-associated DEATH domain protein) inhibits the activation of caspases [45]. BIRC2 (baculoviral IAP repeat containing) and BIRC3 are also known mediators of apoptosis suppression which also interact with the TNF-mediated activation of caspases [46]. These data suggest that the decrease in cell death, observed in CB-MSCs when cultured in human serum, is due to a suppression of TNF-mediated activation of apoptosis. Accordingly, the PCR data also indicated that further downstream in the mitochondrial apoptosis pathway, one of the important mediators, APAF1 (apoptotic protease activating factor 1), is differentially expressed between FBS and human serum, with higher expressions post-AcS in FBS-treated CB-MSCs [47].

## 4. Limitations

Clearly, our *in vitro* hypoxia/reoxygenation model mirrors the situation in myocardial ischemia incompletely, but cell behavior cannot be directly observed in respective *in vivo* experimental models. The culture period of 5 days is short; in cell products requiring longer MSC expansion, the impact of the different sera may differ. We chose this period because we were interested in primary CB-MSC proliferative behavior and stress response. Because cells usually reached confluence at day 5, passaging/subcultivation would be required for longer periods, impeding direct readouts regarding cell number and so on. The average serum donor age differed by roughly 10 years between the CS and HFS groups. This may have impacted the biologic activity of the sera. However, given that both groups were more or less middle-aged while the cells were neonatal, this confounding factor should be of little relevance. Finally, it may be argued that FBS is a pooled product, while the human sera were used individually. We chose not to pool the human sera because we were interested in the heterogeneity of serum activity and because in the clinical setting, autologous human serum would obviously not be pooled. Taken together, we feel that our data support the concept of using autologous serum for cardiac cell therapy, but other experimental designs would be required to understand biology and therapeutic relevance in greater detail.

## 5. Conclusions

At odds with current clinical practice, human serum for MSC expansion in 2D culture is not only equivalent but also may even be superior to FBS in terms of proliferative capacity and resilience to acute and chronic “ischemic” stress. This is also the case when potentially autologous serum from patients with advanced heart failure is used. Using autologous serum simplifies GMP grade translational cell expansion, ought to reduce costs, avoids potential problems with xenogenic biomolecules, and may even have positive preconditioning effects on therapeutic cells.

## Figures and Tables

**Table 1:** Clinical trials investigating the therapeutic potential of MSCs in cardiovascular disease within the past five years. BM-MCSs: bone marrow mesenchymal stem cells; BM-MNCs: bone marrow mononuclear cells; FBS: fetal bovine serum; UC-MCSs: umbilical cord mesenchymal stem cells; SVF: stromal vascular fraction; A-MSCs: adipose tissue-derived mesenchymal stem cells; CHF: congestive heart failure; AMI: acute myocardial infarction; CAD: coronary artery disease without clinical signs of AMI.

Registry identifier	PMID of publications	Trial name/first author	Cell types	Medium supplementation	CV disease treated
NCT01768702	26662998	CHART-1	Autologous BM-MSCs, exposed to “cardiogenic cocktail”	Pooled human platelet lysate	CHF
NCT00810238	23583246	C-CURE	Autologous BM-MSCs, exposed to “cardiogenic cocktail”	Pooled human platelet lysate	CHF
NCT02323477	26123356	HUC-HEART	Umbilical cord MSCs	Pooled human AB serum	AMI
NCT00721045	26148930	Perin et al.	Autologous STRO-1 immunoselected BM-MSCs	FBS	CHF
NCT00644410	25926562	MSC-HF	Autologous BM-MSCs	FBS	CHF
NCT00883727	25484310	Chullikana et al.	Allogeneic pooled BM-MSCs	FBS	AMI
NCT01392105	24431901	Lee et al.	Autologous BM-MSCs	FBS	AMI
NCT01291329	26162993	Gao et al.	Allogeneic UC-MSCs	FBS	AMI
—	24975729	Wang et al.	Autologous BM-MSCs	FBS	AMI
NCT00768066	24247587	TAC-HFT	Autologous BM-MSCs	FBS	CHF
NTR1553 (Dutch trial registry)	23982478	Rodrigo et al.	Autologous BM-MSCs	FBS	AMI
ChiCTR-TRC-08000080 (Chinese clinical trial registry)	23651816	Gao et al.	Autologous BM-MSCs	FBS	AMI
NCT00587990	24565698	PROMETHEUS	Autologous BM-MSCs	FBS	CAD
NCT01087996	23117550	POSEIDON	Autologous and allogeneic BM-MSCs	FBS	CHF
NCT01392625	27856208	POSEIDON-DCM	Autologous and allogeneic BM-MSCs	FBS	CHF
NCT02467387	27856497	Butler et al.	“Ischemia tolerated” BM-MSCs	FBS	CHF
NCT00260338	24211066	Mathiasen et al.	Autologous BM-MSCs	FBS	CAD
NCT00418418	25797522	Lethinen et al.	BM-MNCs	No *in vitro* expansion	CHF
NCT01299324	26217065	REVIVE	Autologous BM-MNCs	No *in vitro* expansion	CHF
NCT01033617	23265095	IMPACT-CABG	Autologous CD133^+^ BM-MNCs	No *in vitro* expansion	CAD
NCT00824005	22447880	FOCUS-CC TRN	BM-MNCs	No *in vitro* expansion	CHF
NCT00684021	23129008	TIME	BM-MNCs	No *in vitro* expansion	AMI
NCT00395811	25418212	Qi et al.	Autologous BM-MNCs	No *in vitro* expansion	CHF
NCT01502514	27255774	Parcero et al.	SVF	No *in vitro* expansion	CHF
NCT02052427	27148802	ATHENA I/II	SVF (A-MSCs)	No *in vitro* expansion	CHF
NCT00426868	24952864	PRECISE	SVF (A-MSCs)	No *in vitro* expansion	CHF
NCT01670981	27059887	ixCELL-DCM	“Ixmyelocel-T” from BM-MNCs	Proprietary culture system; supplement not disclosed	CHF
NCT01076920	26901787	MESAMI 1	Autologous BM-MSCs	Supplement not described	CHF

**Table 2:** Clinical characteristics of serum donors. Where applicable, data are shown as mean ± SEM. MI: myocardial infarction; CAD: coronary artery disease; NYHA: New York Heart Association functional classification; LVEDD: left ventricular end-diastolic diameter; LVEF: left ventricular ejection fraction. ^∗^*p* < 0.05 compared to healthy volunteers.

	Healthy volunteers (*n* = 12)	Heart failure patients (*n* = 12)
History		
Age (years)	54 ± 1.60	64 ± 3.26^∗^
Gender (male/female)	7/5	9/3
Smoker	2	2
Ex-smoker	3	10
Previous MI	—	8
Time since most recent MI (years)	—	8 ± 3.39
Extent of CAD		
Two vessels	—	2
Three vessels	—	10
NYHA functional class		
III	—	5
IV	—	7
Echocardiogram		
LVEDD (mm)	—	65 ± 2.73
LVEF (%)	—	22 ± 2.06
Pulmonary hypertension	—	3
